# Prevalence and predictors of anxiety, depression, and suicidal ideation among Namibian public university students

**DOI:** 10.4102/sajpsychiatry.v31i0.2590

**Published:** 2025-12-18

**Authors:** Kristine N. Siseho, Roswitha Mahalie, Tuwilika Endjala

**Affiliations:** 1 Department of Health Sciences, Faculty of Health, Natural Resources and Applied Sciences, Namibia University of Science and Technology, Windhoek, Namibia; 2 Department of Preventative Health Sciences, School of Health Sciences, Faculty of Health, Natural Resources and Applied Sciences, Namibia University of Science and Technology, Windhoek, Namibia; 3 Department of Community and Mental Health, School of Nursing and Public Health, University of Namibia, Windhoek, Namibia

**Keywords:** anxiety, depression, suicidal ideation, student

## Abstract

**Background:**

Anxiety, depression, and suicidal ideation are prevalent mental health conditions among university students. Persistent anxiety and depression are associated with morbidity and suicidal ideation predicts suicide. Namibian mental health data are limited.

**Aim:**

This study aimed to determine and analyse the prevalence and predictors of anxiety, depression, and suicidal ideation among Namibian public university students.

**Setting:**

The study was conducted at six campuses of the two Namibian public universities.

**Methods:**

A quantitative, cross-sectional, descriptive study was conducted among 578 purposively sampled students, using self-administered questionnaire, incorporating Beck’s Anxiety Inventory, Patient Health Questionnaire-9, and Columbia Suicide Severity Rating Scale. Data were analysed in SPSS version 30, including descriptive statistics, bivariate regression, Pearson’s correlation coefficients, and hierarchical multiple regression.

**Results:**

Among 578 respondents, 50.7% reported moderate to severe anxiety, 73.2% met depression criteria, and 9.1% reported an active suicidal plan. Current mental illness was significantly associated with depression and suicidal ideation (*p* < 0.001). Depression was predicted by current mental illness (*β* = 0.276, *p* < 0.001) and family conflict (*β* = –0.144, *p* < 0.001), while suicidal ideation was predicted by current mental illness (*β* = –0.198, *p* < 0.001), (*p* < 0.001), family conflict (*β* = –0.171, *p* < 0.001), and age (*β* = –0.103, *p* = 0.007).

**Conclusion:**

Anxiety, depression, and suicidal ideation are highly prevalent among Namibian university students, necessitating increased mental health awareness and institutional interventions to prevent suicidality.

**Contribution:**

This study provides empirical evidence on the anxiety, depression, and suicidal ideation among Namibian public university students.

## Introduction

Mental health disorders are the most significant contributors to the global burden of diseases, with anxiety and depression ranking among the foremost challenges in public health.^1^ Transitioning into higher education is a stressful experience for many students.^2^ Factors associated with anxiety, depression, and suicidal ideation (ADSI) include academic stress, increased social media use, female gender, exposure to traumatic events, sexual violence, loss of loved ones, and pre-existing mental health treatment.^3,4^ Globally, 970 million people experience mental health disorders, affecting one in eight individuals, including 58 million children and teens.^5^ Anxiety and depression are linked to suicidal ideation and attempts, as any psychiatric diagnosis increases the risk.^6^ If untreated, depression can lead to suicide, claiming thousands of lives each year.^7^ The World Health Organization reported that each year, over 727 000 people die by suicide worldwide.^8^ Any death by suicide is a painful event for close relatives and friends, prompting many to question what could have been done to prevent such an outcome.^9^ Approximately 332 million people in the world have depression, which is 1.5 times more common in women than in men.^10^ In addition, 358 million people live with anxiety disorders, making anxiety the most common of all mental disorders.

In the Nigerian context, ‘depression has been a significant predictor of suicidal ideation among undergraduates at Enugu State University’.^11^ However, in South Africa, 33.5% of university students reported experiencing depression, while 20.9% reported anxiety symptoms.^12^ Namibia’s mental health policy does not adequately address the nature and severity of mental health problems, especially among university students.^13^ University students are a high-risk group for mental health challenges, with most psychiatric disorders developing by age 24.^14^ To date, there is a lack of data from Namibia on the prevalence and predictors of ADSI among undergraduate students, specifically focusing on second- and third-year students aged 18–24 years. The study hypothesised that gender, age, and educational level would be associated with ADSI. Additionally, personal traits such as higher self-esteem and good social support are linked to resilience against anxiety and depression. Meanwhile, a history of mental illness, academic factors, maltreatment, substance abuse, and social support were predictors of ADSI. Therefore, the aim of this study was to determine the prevalence and predictors of anxiety, depression, and suicidal ideation among Namibian public university students.

## Research methods and design

### Study design

A quantitative research design was utilised employing a cross-sectional, descriptive design to establish and analyse the predictors and correlates of ADSI among university students in Namibia. The participants completed the self-administered questionnaire that gathered the demographic information and predictive factors, including academic, lifestyle, current mental illness, personality, alcohol and substance abuse, depression, and anxiety symptoms, including a binary enquiry of suicidal ideation. A sampling frame consisted of UNAM and NUST campuses, being the only two public universities in Namibia. Two NUST main and Eenhana campuses were purposively selected as they represent NUST sites nationwide. For UNAM, a systematic simple random sampling method was applied: from a list of all 12 campuses, every fourth campus was selected, ensuring equal probability of inclusion. This yielded four UNAM campuses: UNAM main and Hage Geingob (Khomas region, Central), Southern campus (Southern campus in //Karas region), and Oshakati campus (Oshana region, North). To test the research hypothesis, a structured questionnaire was distributed in person to respondents to collect relevant data on the main predictors of ADSI among undergraduate students in Namibia.

### Setting

Data were collected from NUST and UNAM multiple campuses across Namibia, including in the Khomas, Oshana, Ohangwena, and Omusati regions. These regions were selected based on suicide prevalence: Oshana, Ohangwena, and Omusati ranked among the top five with the highest suicide rates, while the Khomas and //Karas regions have the lowest suicide rate in Namibia.^15^

### Study population and sampling strategy

The study population included undergraduate students aged 18–24 years, specifically those in their second and third academic years who volunteered to participate. The self-administered questionnaires were distributed to 780 participants recruited through a purposive sampling technique, which lasted for a period of 2 months.^16^ The response rate was 83%, representing 578 completed questionnaires from which the study findings were drawn. The sample size was calculated using Yamane’s formula (1973), *n* = *N* ÷ (1 + *N* × e^2^), where N = population size, 1 = a constant, and e = margin of error, set at 0.05 or the significance level.^15^

### Data collection and instrument

The self-administered questionnaires were designed in English and consisted of three sections. The first section, Section A, included demographic items targeting respondents aged 18–24 years who are current undergraduates in their second and third academic years from the participating campuses. Demographic data were collected from the respondents. Section B contained predictors associated with ADSI among respondents. The hypothesis included economic, family, and academic factors. Section C examined symptoms of anxiety and depression, as well as the evidence of suicidal ideation, which contributed to the prevalence of ADSI among public university students in Namibia. The questionnaires were handed to the participants in person.

### Patient health questionnaire-9

The Patient Health Questionnaire-9 (PHQ-9) is a widely used, validated tool to assess depression, consisting of nine items that measure the frequency of depressive symptoms over a specific period.^17^ Patient Health Questionnaire-9 was used to screen for depression symptoms among the participants. Depression symptoms listed in DSM-5 include: (1) lack of interest, (2) depressed mood, (3) sleeping disturbances, (4) reduced energy levels, (5) eating disorders, (6) negative feelings about oneself, (7) psychomotor retardation, and (8) difficulty in concentrating.^18^ Respondents were instructed to rate symptoms on a 4-point Likert scale, indicating how often they experienced depression symptoms in the past two weeks (0 = not at all, 1 = several days, 2 = more than half the days, and 3 = every day). Scores for each category ranged from 0 (never) to 3 (every day). The PHQ-9 scores ranged from 0 to 27 and were categorised as follows: 0–7 indicating none to minimal depression symptoms, 8–16 mild depression, 17–24 moderate depression, and 25–27 severe depression. The Cronbach’s α coefficient was 0.874 for depression, indicating strong internal consistency.

### Beck’s Anxiety Inventory

The study modified Beck’s Anxiety Inventory (BAI) sheet, which included seven items used to determine the prevalence of anxiety symptoms among university students.^19^ The seven items used a four-point Likert scale, where 0–4 indicates minimal anxiety symptoms, 5–9 indicates mild anxiety symptoms, 10–14 indicates moderate anxiety, and 15–21 indicates severe anxiety. The internal consistency was confirmed by Cronbach’s α coefficient of 0.805, indicating strong internal consistency.

### Suicidal ideation

Suicidal ideation was assessed using the Columbia Suicide Severity Rating Scale Version 14 January 2009.^20^ The scale included binary questions: ‘Yes’ for those who are suicidal, and ‘No’ for non-suicidal individuals. One score indicates the lowest level, while five represents severe suicidal ideation. The open-ended questionnaire asked participants to describe how they would like to die, to evaluate suicidal ideation in the past 2 weeks. The Cronbach’s alpha was 0.726, indicating an acceptable reliability.

### Data analysis

Descriptive statistics, including frequencies and percentages, were used to summarise the demographic characteristics of the respondents. Descriptive statistics were analysed in SPSS version 30.^21^ To understand the correlation between anxiety, depression, and suicidal ideation, Pearson’s correlation coefficients were calculated. Hierarchical multiple regression analysis was performed to identify predictors of ADSI. Binary logistic regression was used to determine the prevalence of suicidal ideation categorised as ‘Yes’ or ‘No’. A *p*-value < 0.05 was considered statistically significant. All variables, including demographic characteristics, prevalence, and predictors, were analysed to identify independent predictors of ADSI among students.

### Ethical considerations

The relevant institutions granted permission to conduct the study. Ethical approval was obtained from the Namibia University of Science and Technology Research Committee, with reference number FHNRAS:60/2023, and from the Namibian Ministry of Health and Social Services, with reference number 22/3/1/2. The six campuses where the study was conducted approved the data collection. The study adhered to ethical standards for health science research. In this quantitative study, steps were taken to ensure anonymity, voluntary participation, the right to withdraw, and respondent confidentiality. The research tool’s cover page included an informed consent form, which participants signed before the data collection began. The researcher collected data using self-administered questionnaires distributed to students who met the inclusion criteria, were available, and gave their consent to participate during scheduled campus visits. No emotional distress was observed among participants during the study. However, had any such responses emerged, appropriate referrals for counselling would have been initiated.

## Results

### Socio-demographic characteristics of respondents

The data set included a total of 578 undergraduates from NUST and its Eenhana satellite campus, as well as the UNAM main campus, Hage Geingob campus, Oshakati campus, and Southern campus. Second- and third-year students enrolled at public universities were recruited for the study’s survey (Table 1). Most respondents were female, at 55.4%, while 44.6% were male. They consisted of 53.6% second-year students and 45.4% third-year students. Among the respondents, 25.3% lived in the campus hostel, and 72.9% lived off campus.

**TABLE 1:** Socio-demographic characteristics of study participants (***N*** = 578).

Demographic variables	Category	*n*	%
**Age (years)**	18–19	66	11.4
	20–24	512	88.6
**Gender**	Male	258	44.6
	Female	320	55.4
**Academic year**	Second	310	53.6
	Third	262	45.3
**Residence**	Campus hostel	146	25.3
	Commute – Urban home	253	43.8
	Commute – Rural home	38	6.6
	Informal settlement	51	8.8
	Other	79	13.7
	Did not answer	11	1.9

### The prevalence of anxiety and depression among the students

The prevalence of anxiety and depression symptoms was determined using frequencies and percentages to assess the extent of these conditions among respondents. As shown in Table 2, 43.3% experienced mild anxiety symptoms, 42.6% had moderate anxiety symptoms, and 8.1% exhibited severe anxiety symptoms. Regarding depression, 21.8% reported mild symptoms, 43.1% moderate symptoms, and 30.1% had severe depression symptoms.

**TABLE 2:** Frequency of the prevalence of anxiety and depression (***N*** = 578).

Symptom level	Anxiety symptoms		Depression symptoms	
	*n*	%	*n*	%
Minimal	35	6.1	29	5.0
Mild	250	43.3	126	21.8
Moderate	246	42.6	249	43.1
Severe	47	8.1	174	30.1

**Total**	**578**	**100.0**	**578.0**	**100.0**

### Prevalence of suicidal ideation

The findings reveal various types of suicidal ideation among university students who answered ‘Yes’ (Table 3): passive suicidal ideation, 25.1%; suicidal ideation without a plan, 19.9%; active suicidal ideation, 15.2%; and suicidal thoughts in general, 13.5%. Alarmingly, active suicidal ideation with a plan was 9.7%. Although most participants responded ‘No’ across all variables, it is essential to recognise that ‘Yes’ indicates a significant mental health concern among students.

**TABLE 3:** Frequency and percentage of suicidal ideation.

Variable	Response	*n*	%
Passive suicidal ideation	Yes	145	25.1
	No	432	74.9
Suicidal ideation without a plan	Yes	115	19.9
	No	463	80.1
Active suicidal ideation	Yes	88	15.2
	No	490	84.8
Suicidal thoughts (general)	Yes	80	13.8
	No	498	498
Active suicidal ideation with a plan	Yes	56	9.7
	No	517	89.4

### Pearson’s correlational coefficient analysis of anxiety, depression, and suicidal ideation

The research aimed to establish the correlation between ADSI and students. Pearson’s correlation coefficient was calculated to explore the relationships between anxiety, depression, and suicidal ideation (Table 4). There was a positive correlation between anxiety and depression (*r* = 284, *p* < 0.001), and between anxiety and suicidal ideation (*r* = 166, *p* < 0.001). Similarly, depression significantly correlated with suicidal ideation (*r* = 0.362, *p* < 0.001). Current mental illness was positively associated with anxiety (*r* = 0.162, *p* < 0.001), depression (*r* = 0.276, *p* < 0.001), and suicidal ideation (*r* = 0.261, *p* < 0.001). Family conflict showed a weaker but significant positive correlation with anxiety (*r* = 0.088, *p* < 0.034), depression (*r* = 111, *p* < 0.007), and suicidal ideation (*r* = 0.117, *p* = 0.004), while a weak but statistically significant negative correlation was observed with suicidal ideation (*r* = –0.111, *p* = 0.007) indicating that resilience has a protective influence.

**TABLE 4:** Correlation table for anxiety, depression, and suicidal ideation.

Variable	Anxiety	Depression	Suicidal ideation	Current mental illness	Family conflict
**Anxiety**	1	-	-	-	-
Pearson’s *r*	-	0.284	0 .166	0.162	0.088
*p*-value	-	< 0.001**	< 0.001**	<0 .001**	0.034*
**Depression**	-	1.000	-	-	-
Pearson’s *r*	-	-	0.362	0.276	0.111
*p*-value	-	-	< 0.001**	< 0.001**	0.007*
**Suicidal ideation**	-	-	1.000	-	-
Pearson’s *r*	-	-	-	0.261	0.117
*p*-value	-	-	-	< 0.001**	0.004*
Current mental illness	-	-	-	1.000	-
Family conflict	-	-	-	-	1.000

* , Pearson’s correlation coefficient between anxiety, depression and suicidall ideation.

** , p < 0.05 (not statistically significant), p < 0.01 (not statistically significant), p < 0.001. All correlations are Pearson’s r.

### Predictors of anxiety, depression, and suicidal ideation

A series of hierarchical regressions was conducted to identify the predictors of ADSI among students at public universities. Table 5 shows that the significant predictors of anxiety included current mental illness (*β* = –0.162, *p* < 0.001), personality (*β* = 0.131, *p* = 0.001), and faculty (*β* = 0.077, *p* = 0.031). Significant predictors of depression included current mental illness (*β* = –0.276, *p* < 0.001), family conflict (*β* = –0.144, *p* < 0.001), and personality (*β* = 0.087, *p* = 0.019). For suicidal ideation, significant predictors included current mental illness (*β* = –0.198, *p* < 0.001), faculty (*β* = –0.123, *p* < 0.001), family conflict (*β* = –0.171, *p* < 0.001), and age (*β* = –0.103, *p* = 0.007).

**TABLE 5:** Hierarchical regression: predictors of anxiety, depression, and suicidal ideation.

Predictor	Anxiety (*β*)	*p*-value	Depression (*β*)	*p*-value	Suicidal ideation (*β*)	*p*-value
Age	0.001	0.495	0.016	0.352	−0.103	0.007*
Gender	0.075	0.035*	0.050	0.115	−0.033	0.213
Academic year	−0.003	0.471	−0.053	0.129	−0.097	0.010*
Faculty	0.077	0.031*	−0.047	0.140	−0.123	0.001**
Current mental illness	−0.162	0.001***	−0.276	0.001**	−0.198	0.001**
Personality	0.131	0.001*	0.087	0.019*	0.054	0.096
Family conflict	−0.022	0.302	−0.144	0.000*	−0.171	0.001*
First-year experience	−0.048	0.001*	−0.037	0.141	−0.080	0.032
Feeling after failing a module	0.030	0.234	−0.045	0.141	−0.104	0.006*
Lifestyle smoking	−0.003	0.472	−0.004	0.458	−0.072	0.041
Lifestyle alcohol abuse	0.066	0.057	−0.077	0.032*	−0.062	0.067
Drug abuse	0.052	0.105	0.038	0.181	0.051	0.109

* , *p* < 0.05,

** , *p* < 0.01,

*** , *p* < 0.001.

+*β* = higher predictor – higher the outcome.

−*β* = means higher predictor – lower outcome.

## Discussion

The study identified and analysed the prevalence and predictors of anxiety and depression symptoms and suicidal ideation among Namibian public university students. It also examined the relationship between ADSI, demographic factors, and predictors such as academic year, family conflicts, faculties, current mental health issues, alcohol and substance misuse, gender, and age. Using data from primary sources, the prevalence of ADSI was thoroughly determined. The sample reported higher levels of anxiety and depression symptoms compared with suicidal ideation.

In this study, scientific evidence showed that 50.7% of students reported experiencing moderate to severe anxiety symptoms, and 73% reported having moderate to severe depressive symptoms. These findings were notably higher compared with studies carried out in other countries. The prevalence of anxiety and depressive symptoms in our study exceeded 25%, with depression at 45.3%, compared with a study conducted in Germany.^21,22^ Interestingly, in Ghana, the prevalence of anxiety symptoms was 53.3%, and depression symptoms were 25.2% concurrently.^23^ This study’s findings are attributed to factors such as students migrating from rural areas to universities, academic pressure, and the transition process, highlighting the contribution of these multifaceted factors to ADSI. Concurrent with the findings from a study done in Namibia among the undergraduate student which reported several factors contributes to their mental health challenges. This include fear related to academic transition, adjusting to unfamiliar environment, accommodation, financial challenges, family conflicts and academic pressure.^23^ These factors can affect the student’s mental stability, thereby affecting their academic performance.

Assessment of suicidal ideation revealed that some students experienced active thoughts of suicide, with 9.7% of them reporting a specific plan to carry it out. This study’s findings contrast with the Nigerian study, which found a much higher rate; 85.2% of students had passive suicidal ideation compared with active ideation. In this study, multiple predictors of suicidal ideation were identified, including age, academic year, faculty of study, current mental illnesses, family conflicts, and educational failure. The predictors of suicidal ideation in Nigeria included academic challenges, substance use, family conflicts, and other psychological stressors, which increase vulnerability among undergraduates.^24^ The transitioning from suicidal ideation to suicide attempt is recognised as a clear occurrence characterised by unique factors and predictors that were beyond the scope of investigation in this study.^25^

Previous mental illness is a predictor of ADSI, consistent with a study in Sudan, indicating that a prior psychiatric diagnosis of general anxiety disorder (GAD) corresponds to the prevalence of GAD, which ranges from 4% to 7%.^25^ Alcohol was identified as a predictor of anxiety, consistent with a study that found social anxiety to be associated with alcohol use.^26,27^ Meanwhile, participants in this study identified rising alcohol consumption among students as a matter of concern. Academic pressure increases over the years, as the study predicts that the academic year correlates with suicidal ideation, which can be linked to students contemplating their careers and the competitive job market. In Bangladesh, for instance, higher unemployment has been identified as a contributing factor to student stress.^28^ Similarly, a 2018 Namibian survey found that the unemployment rate among youth aged 15–35 years was 46.1%.^29^ These socio-economic pressures compound the academic and psychological stress faced by students, potentially leading to poor mental health outcomes and suicidality. A systematic review in Africa of people living with mental illness showed that the quality of life (QoL) was 45.93% (95% CI = 36.04, 55.3), reflecting poor QoL among adults with mental illness.^30^ The prevalence of depression, anxiety, and stress is significantly higher in lower and middle-income countries, including Namibia. Among Chinese students, self-esteem is also a predictor of anxiety, which makes an individual prone to negative self-evaluation, feelings of worthlessness, and increased negative sensations, leading to anxiety and discomfort.^31^ The study did not investigate the association between age, gender, or academic year and the development of ADSI symptoms. However, females are typically more likely to present with depression and twice as likely to suffer from anxiety and suicidal attempts at an earlier age than males who suffer from depression.^32^

The study’s analysis showed that the faculty of study had no significant effect, implying that age and academic year were negatively linked to suicidal ideation, suggesting that maturity and academic progress might offer protective benefits.^33^ This research highlights the need for further investigation to clarify these associations.

This study was limited to only two public universities in Namibia, with the sample predominantly composed of females, which may affect the generalisability of the study’s findings. The inclusion of only two NUST campuses, compared with broader representation from UNAM, may also influence the prevalence estimates of anxiety, depression, and suicidal ideation per campus. Moreover, the cross-sectional design did not assess clinical diagnosis but rather the symptoms of anxiety and depression. Future longitudinal research is recommended to examine the impact of ADSI on academic performance across diverse age groups, postgraduate cohorts, and institutions to better understand the resilience throughout a student’s academic journey.

## Conclusion

This study’s findings reveal the silent mental health crisis developing among university students, characterised by high rates of anxiety, depression, and suicidal ideation. Although the university provides counselling support for students experiencing ADSI, there is room for improvement, which may involve incorporating the screening tool for early detection and treatment of these conditions. This emphasises that mental illness can no longer be regarded as a peripheral issue. Public university mental health providers must urgently shift from a reactive approach to a proactive one that identifies, supports, and empowers students before a crisis occurs. Addressing student mental health requires immediate and ongoing action, as it is essential for students to succeed amid the pressures of academic demands and socio-economic uncertainty.
